# Impacts of Lead and Nanoplastic Co-Exposure on Decomposition, Microbial Diversity, and Community Assembly Mechanisms in Karst Riverine *Miscanthus* Litter

**DOI:** 10.3390/microorganisms13092172

**Published:** 2025-09-17

**Authors:** Peijian Chen, Tianjiao Mei, Xingbing He, Yonghui Lin, Zaihua He, Xiangshi Kong

**Affiliations:** 1 College of Biology and Environmental Sciences, Jishou University, Jishou 416000, China; chenpeijian6078@163.com (P.C.); mtj2025@163.com (T.M.);; 2 Hunan Provincial Key Laboratory of Ecological Conservation and Sustainable Utilization of Wulingshan Resources, Jishou University, Jishou 416000, China; 3 College of Tourism and Management Engineering, Jishou University, Zhangjiajie 427000, China; kongxiangshi@126.com

**Keywords:** lead, nanoplastic, mass loss, microbial diversity, microbial community assembly, microbial co-occurrence network

## Abstract

Karst rivers are increasingly contaminated by both heavy metals and nanoplastics, yet their combined impact on riparian litter decomposition remains unresolved. We conducted a 90-day microcosm experiment using *Miscanthus floridulus* leaf litter collected from the Donghe River, Jishou, China, and exposed it to Pb (1 mg L^−1^), polystyrene nanoplastics (10 and 100 µg L^−1^), and their combinations. Pb alone modestly inhibited mass loss (61.0%) and respiration, while NP10 significantly accelerated decomposition (67.0%), and NP100 suppressed it (60.4%); co-exposure produced non-monotonic, concentration-dependent effects. Enzyme stoichiometry revealed that all treatments intensified nitrogen limitation but alleviated carbon limitation through reduced microbial activity. Bacterial communities, dominated by *Pseudomonadota*, exhibited remarkably stable phylum-level composition, high network complexity, and identical keystone taxa across all treatments, indicating strong functional redundancy and resilience. In contrast, fungal communities suffered severe declines in *Basidiomycota* abundance, collapsed network stability, and a single keystone taxon, underscoring their vulnerability. βNTI–RC_bray_ analyses demonstrated that stochastic processes (>50%) overwhelmingly governed both bacterial and fungal assembly, with only marginal deterministic shifts. Collectively, our findings highlight that bacteria—not fungi—serve as the primary decomposers under Pb–NP co-stress and that stochastic assembly, coupled with bacterial redundancy, buffers ecosystem function against emerging mixed pollutants in subtropical riverine systems.

## 1. Introduction

Fluvial ecosystems provide a multitude of ecosystem services directly linked to their functioning [1]. Annually, riverine ecosystems contribute to global carbon cycling by mineralizing immense quantities of allochthonous riparian leaf litter, which is an essential energy resource in aquatic food web, thereby regulating downstream fluxes of carbon, nitrogen, and phosphorus on a global scale [2,3,4]. Recent syntheses estimate that tropical, temperate, and high-latitude rivers collectively emit 1.9–2.3 Pg of carbon per year, a magnitude equivalent to approximately 37% of the terrestrial organic carbon flux as CO_2_ [5]. The process is governed by extracellular enzymes (β-glucosidase, phenol oxidase, cellobiohydrolase) secreted by microbial consortia dominated by *Proteobacteria*, *Actinobacteria* and ligninolytic *Basidiomycota* [6,7]. River networks are also vital human settlement areas, and their ecological processes, including litter decomposition, are inevitably dominated by anthropogenic activities [3]. Consequently, investigating plant litter decomposition and its associated microbial community composition in riverine ecosystems is of critical importance for gauging ecosystem stability.

Among riparian plants, the C_4_ perennial grass *Miscanthus floridulus* has become a dominant component of flood-plain vegetation across subtropical and temperate rivers. Owing to its vigorous clonal expansion, rapid growth and high reproductive output, it frequently forms mono-dominant stands on mountain slopes, flood-plains and forest margins. These dense thickets intercept rainfall, enhance infiltration and stabilize soils, reducing surface runoff and landslide risk; they therefore provide critical erosion-control and water-retention services. The species also yields exceptional biomass (20–40 t ha^−1^ yr^−1^), making it one of the most important living carbon sinks in riparian corridors [8]. *Miscanthus* litter represents a persistent energy source for benthic detritivores and microbial decomposers, and it modulates downstream particulate organic-carbon fluxes through tight “bank-to-channel” coupling. Despite the quantitative significance of this litter pool, its decomposition dynamics under the increasingly common co-occurrence of lead (Pb) and nanoplastics (NPs) remain virtually unknown. Heavy-metal contamination has repeatedly been shown to decelerate litter breakdown [9]. For instance, Cd, Cu, Zn addition generally reduced litter decomposition by inhibiting cellulose-degrading enzyme activities of β-1,4-glucosidase and cellobiohydrolase, and lignin-degrading enzyme activities of phenol oxidase and peroxidase [10,11,12,13,14]. Chen et al. [15] also reported that Cd led to a significant decrease in litter decomposition rate, with a maximum decrease of 32.1% in *Solanum nigrum* and 30.1% in *S*. *lycopersicum*. Song et al. [16] also revealed a decline of 46.3% of soil CO_2_ emission by Pb addition in a field experiment. Beyond metals, plastic particles also exert significant ecological impacts. NPs readily adsorb onto biotic or abiotic surfaces; owing to their minute size, NPs are especially prone to entering plant tissues or microbial cells, thereby exerting chronic effects on ecological processes—particularly litter decomposition [17,18]. Du et al. [19] reported that chronic exposure to NPs at 1–100 μg L^−1^ markedly decreased *Populus nigra* leaf-litter decomposition and nutrient release in a simulated freshwater system. Their follow-up work further demonstrated that NPs suppress litter breakdown by inhibiting fungal reproduction and diminishing the contributions of key fungal taxa [20]. Collectively, these findings underscore that individual or combined stressors can propagate through enzymatic pathways or shifts in microbial community composition, ultimately altering the rate and mode of carbon turnover in fluvial systems.

Microbial diversity, along with the ecological processes that generate it, underpins the stability of stream ecosystems. Alpha-diversity sets the upper limit on metabolic versatility, whereas beta-diversity governs the spatial insurance of functions; together, they regulate the rate and stoichiometry of carbon and nutrient release from decomposing litter. These functions are further modulated by networks of direct (competition, mutualism) and indirect (cross-feeding, metabolite signaling) microbe–microbe interactions that confer resilience to environmental disturbance. Aquatic decomposers, especially bacteria and fungi, are acutely sensitive to physicochemical stressors [21,22,23]. Heavy-metal contamination consistently erodes this biological foundation, reducing microbial biomass and overall diversity [16,24,25]. In a Chinese copper tailings watershed, Jia et al. [26] reported that Pb, Zn, Cu and Cd jointly restructured bacterial communities. Similarly, Zeng et al. [27] observed that Pb–Zn mining effluent decreased bacterial richness but paradoxically increased fungal richness, suggesting divergent tolerance strategies between kingdoms. Metal-tolerant taxa were selectively enriched: *Acidobacteriales*, *Gaiellales*, *Anaerolineaceae*, *Sulfurifustis* and *Gemmatimonadaceae* among bacteria, and *Sordariomycetes*, *Talaromyces* and *Mortierella* among fungi. Xu et al. [28] corroborated that Zn–Pb pollution distinctly altered the abundance of multiple bacterial phyla and genera. Ma et al. [29] further showed a pollution-induced rise in *Proteobacteria* and a concomitant decline in *Actinobacteria*. At 10 mg kg^−1^ Cd, Xiao et al. [30] recorded marked shifts in bacterial and fungal α-diversity, a reduction in *Ascomycota* and *Basidiomycota*, and enhanced soil organic-carbon accumulation. NPs impose an analogous, but mechanistically distinct, selective pressure. Polystyrene NPs restructured rhizosphere bacterial and fungal diversity, composition and network topology [31]. Liu et al. [32] found that NPs exerted stronger effects on prokaryotic than on eukaryotic communities. Zhao et al. [33] documented a 17.7% decline in nitrifying bacteria and a 5.2% increase in *Polaromonas* following repeated NPs pulses. At functional-guild resolution, Hao et al. [34] observed a 1.57-fold enhancement of denitrification under Polystyrene NPs, accompanied by a pronounced shift in the nirS-type denitrifier community. When metals and NPs co-occur, their impacts on microbial communities may be antagonistic, additive or synergistic, yet the net outcome remains poorly resolved.

Community assembly—the suite of processes that generates microbial diversity—has been formalized into five non-exclusive categories: (i) homogeneous selection; (ii) heterogeneous selection; (iii) dispersal limitation; (iv) ecological drift; and (v) homogeneous dispersal [35]. Quantifying the relative contribution of each process relies on null-model metrics such as the β-nearest taxon index (βNTI) and the modified Raup–Crick metric (RC_bray_). It is widely accepted that environmental stressors impose selective pressures on microbial diversity and co-occurrence networks, thereby shifting the balance among assembly processes [36]. Heavy-metal contamination is a well-established driver of microbial community assembly. Li et al. [31] reported that combined metal exposure strengthened bacterial network stability and complexity, whereas fungal networks were markedly simplified, losing 33.7% of their positive edges. In another study, Li et al. [37] observed a pollution-induced decline in overall microbial diversity that was accompanied by reduced network complexity and elevated modularity; assembly analyses revealed a deterministic shift in bacterial communities under high pollution, while fungal assemblages remained predominantly stochastic. Extending these findings, Wang et al. [38] showed that Se and multi-metal pollution fragmented microbial networks in contaminated soils. In control soils, stochastic drift dominated assembly, whereas dispersal limitation became the primary driver under Se stress, attributable to metal-toxicity barriers. NPs elicit comparable, yet mechanistically distinct, selective pressures. Guan et al. [39] demonstrated that single-pulse NP exposure reduced network complexity in seawater communities. When polystyrene NPs concentrations reached 1000 μg L^−1^ over 87–132 days, removal efficiencies for chemical oxygen demand, total phosphorus and total nitrogen fell by 2.7%, 33.2% and 23.5%, respectively [40]. These functional losses coincided with topological degradation: networks became smaller, less complex and exhibited weaker material-transfer efficiency. Crucially, subnetworks dedicated to nitrification and nitrogen removal were disproportionately suppressed, resulting in elevated effluent NH_4_^+^-N and TN. Co-occurrence analyses further revealed that NPs amendments fostered a denser denitrifier network and restructured keystone taxa; *Sideroxydans* increasingly cooperated with *Burkholderiales* to sustain denitrification [34]. Finally, Zhang et al. [41] demonstrated that polypropylene NPs diminished bacterial network complexity and connectivity, shifting the dominant assembly process from stochastic drift to deterministic selection.

These converging lines of evidence demonstrate that both Pb and NPs can independently—and potentially interactively—reshape microbial diversity and community assembly. However, a critical knowledge gap remains at the intersection of ecotoxicology and ecosystem ecology. No study has yet integrated these processes with the decomposition kinetics of Miscanthus litter under environmentally realistic co-exposure scenarios. We therefore tested whether Pb and NPs interact synergistically to suppress Miscanthus litter decomposition, while also reshaping microbial diversity and altering community assembly rules. We hypothesized that (H1) Pb–NP co-exposure will inhibit litter decomposition and microbial activities compared with the control; (H2) co-exposure will decrease bacterial and fungal α-diversity, and change microbial composition; and (H3) co-exposure will strengthen deterministic assembly by intensifying homogeneous environmental filtering, thereby reducing the relative influence of ecological drift. The study provides the first mechanistic link between Pb–NP interactions and enzyme-mediated decomposition, forecasts how co-pollution alters riverine carbon turnover and informs sustainable management of *Miscanthus* riparian buffers.

## 2. Materials and Methods

### 2.1. Site Description and Litter Collection

The experimental site is situated on the floodplain of the Donghe River, belonging to a karst system, near A’na Village (28°15′41.51″ N, 109°46′55.61″ E) in Jishou City. Donghe River is an upper tributary of the Yuanjiang River, which itself is a tributary of Dongting Lake within the Yangtze River Basin. This region is characterized by a subtropical monsoon climate, with an average annual temperature ranging from 12 °C to 17 °C and an annual precipitation of 1100 to 1600 mm. The riparian vegetation in this area is dominated by *Miscanthus floridulus* and *Pterocarya stenoptera*. For this study, we selected *M*. *floridulus*—a species widely distributed in subtropical regions with significant ecological roles, such as contributing to soil and water conservation, maintaining riparian ecosystem stability, and providing habitat and resources for various organisms.

**Figure 1:**
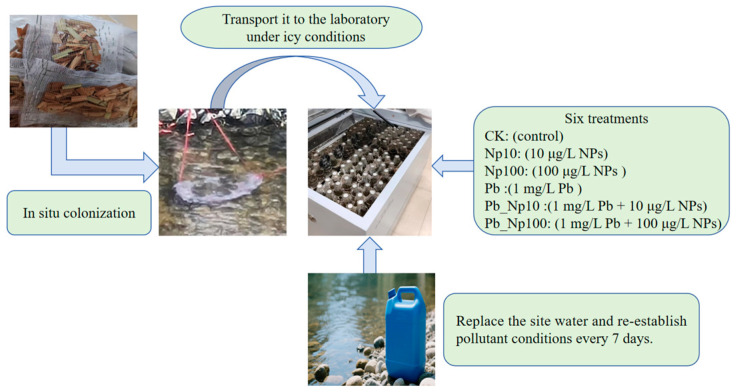
Schematic diagram of the experimental design.

In December 2023, aboveground leaves of *M. floridulus* were collected at the Donghe River site. These leaves served as the substrate for the subsequent litter decomposition experiment, which included two key stages (Figure 1): in situ microbial colonization (conducted at Donghe River) and post-colonization decomposition assay (laboratory experiment described in Section 2.2).

### 2.2. Experimental Design

#### 2.2.1. In Situ Microbial Colonization and Low-Temperature Sample Transport Protocol

For litter pretreatment, Fresh *M*. *floridulus* leaves were cut into 1 cm segments and dried to constant weight in an oven at 50 °C. A 1.5 g dry-weight subsample was placed into sterile nylon litter bags (10 cm × 10 cm, pore size: 0.5 mm). On 18 March 2024, these litter bags were deployed in the surface water of Donghe River (the same collection site mentioned in Section 2.1.) for 10 days to allow in situ microbial colonization. After the completion of in situ colonization, the entire litterbag was placed into a pre-sterilized collection bag. Once the collection bag was sealed, it was transferred into an insulated foam container. A total of 5 kg of ice was evenly distributed inside the insulated foam container, with strict attention to ensuring no direct contact between the ice and the collection bags. Meanwhile, sterile foam dividers were installed between the collection bags and the ice. These dividers served two key purposes: first, to prevent the formation of cold spots inside the container; second, to protect the collection bags from physical damage caused by collision or extrusion during transportation.

A low temperature of about 4 °C was maintained throughout the entire process, and the insulated foam container holding the litterbags was transported back to the laboratory for subsequent further laboratory decomposition.

#### 2.2.2. Laboratory Microcosm Experiment Simulating Natural Conditions for Litter Decomposition

Pollutant stock solution preparation: Monodisperse polystyrene nanoplastics (40–84 nm; Jiangsu Zhichuan Technology Co., Ltd., Nantong, China) were used to prepare three concentration gradients (0, 10, and 100 μg/L). Each suspension was sonicated at 40 kHz for 30 min to achieve homogeneity. The selection of lead concentration was based on the specific environmental context of the Jishou region. Although the protected sections of local rivers currently show Pb levels below 1 μg/L, the area contains the largest lead-zinc deposit in China. Historical intensive mining activities have resulted in numerous tailings ponds, leading to severe soil Pb contamination—17 to 25 times higher than local background value. The bioavailable Pb content in these areas ranges from 26 to 129 mg/kg [42], causing Pb concentrations in rivers near mining sites to frequently exceed 1 mg/L and posing a continuous threat to downstream protected waters. To simulate this realistic high-exposure scenario, lead-contaminated solutions were prepared using analytical-grade lead chloride (Tianjin Shuangchuan Chemical Reagent Factory, Tianjin, China), filtered through a 0.45 μm membrane, and set at two concentration gradients (0 and 1 mg/L).

Experimental design and setup: A 3 × 2 full factorial design was applied, comprising six treatment groups: CK (control), Np10 (10 μg/L nanoplastics), Np100 (100 μg/L nanoplastics), Pb (1 mg/L Pb), Pb_Np10 (1 mg/L Pb + 10 μg/L nanoplastics), and Pb_Np100 (1 mg/L Pb + 100 μg/L nanoplastics). Each treatment included fifteen independent sterile replicates (250 mL conical flasks), each containing one microbially colonized litterbag and 100 mL of sterile-filtered (1.2 μm) raw river water. Pollutants were added according to group specifications, and flasks were vortexed at 300 rpm for 2 min to ensure uniform dispersion.

Environmental simulation and cultivation conditions: All flasks were incubated in a full-automatic constant temperature shaker (Tianjin Labotery Instrument Equipment Co., Ltd., Tianjin, China) under the following parameters calibrated to simulate natural conditions (March–June) in the Donghe River—Temperature: 25 ± 0.5 °C, reflecting the typical surface water temperature range (23–27 °C); Shaking frequency: 125 rpm to simulate gentle flow conditions in shallow floodplain waters, promoting solute exchange without damaging litter or biofilms; Light cycle: 12 h light/12 h dark at 5000 lux, consistent with local natural photoperiods.

Water renewal and sampling: Every 7 days, the water in each flask was completely replaced with freshly collected river water from a fixed site using a siphon system to minimize disturbance to microbial communities. Pollutant concentrations were re-established according to treatment specifications after each water change.

Sampling for endpoint measurements: Fifteen replicates per treatment were sampled for measurement of all variables after 90 days of incubation.

### 2.3. Extracellular Enzyme Activity, Microbial Respiration and Mass Loss

Upon the completion of the incubation period, leaf samples were finely minced and homogenized in buffer using a mortar and pestle. The resulting slurry was centrifuged, and the supernatant was collected for subsequent enzyme assays. The activities of carbon-degrading enzymes—β-glucosidase (βG, EC 3.2.1.21), exocellulase (C1, EC 3.2.1.91), and endocellulase (Cx, EC 3.2.1.4)—were quantified using the 3,5-dinitrosalicylic acid (DNS) method [43]. Phenol oxidase (EC 1.10.3.2) and peroxidase (EC 1.11.1.7) activities were determined spectrophotometrically with L-3,4-dihydroxyphenylalanine (DOPA) as the substrate [44]. For the assays of chitin and phosphorus degradation, β-N-acetylglucosaminidase (NAG, EC 3.2.1.30) and acid phosphatase (AP, EC 3.1.3.2) activities were measured via the p-nitrophenol (pNP) method, using pNP-N-acetyl-β-D-glucosaminide and pNP-phosphate as their respective substrates [45,46]. Proteolytic activity was assessed as leucine aminopeptidase (LAP, EC 3.4.11.1) using L-leucine-p-nitroanilide (Leu-pNA) and the p-nitroaniline (pNA) method [47]. All enzyme activities are reported as μmol product formed g^−1^ dry litter h^−1^, with five replicates per treatment.

Microbial respiration was determined by incubating 0.5 g of leaf litter in sealed flasks at 25 °C in darkness for two days. The CO_2_ evolved was trapped in 0.5 M NaOH and quantified via two-phase titration with 0.05 M HCl [48]. Results are expressed as μmol CO_2_ g^−1^ dry litter d^−1^, with five replicates per treatment.

Litter mass loss was measured by retrieving five litterbags per treatment. Leaves were oven-dried at 50 °C until constant weight was achieved. Mass loss was calculated as the percentage reduction from the initial dry mass.

### 2.4. DNA Extraction, PCR Amplification, and NovaSeq Sequencing

Total DNA was isolated from all samples using the E.Z.N.A.^®^ Soil DNA Kit (Omega Bio-Tek, Norcross, GA, USA), with five biological replicates prepared for both fungal and bacterial amplicon sequencing. DNA concentration and purity were evaluated on a NanoDrop 2000 spectrophotometer (Thermo Fisher Scientific, Waltham, MA, USA), and integrity was verified via 1% agarose gel electrophoresis (BioWest, Madrid, Spain). The ITS1 region of fungal rRNA genes was amplified with primers ITS1F (5′-CTTGGTCATTTAGAGGAAGTAA-3′)/ITS2R (5′-GCTGCGTTCTTCATCGATGC-3′), and the V5–V7 hypervariable region of the bacterial 16S rRNA gene was amplified using primers 799F (5′-AACMGGATTAGATACCCKG-3′)/1193R (5′-ACGTCATCCCCACCTTCC-3′). All PCRs were conducted on a GeneAmp^®^ 9700 PCR System (ABI, Dublin, CA, USA). Library construction and sequencing were carried out following the protocol outlined in Zhao et al. [49]. Final libraries were paired-end sequenced (PE250) on an Illumina NovaSeq platform at BIOZERN Biotech. Co., Ltd. (Shanghai, China).

Raw sequences were subjected to quality control with Trimmomatic V0.39. Overlapping reads were merged using FLASH, and operational taxonomic units (OTUs) were clustered at 97% sequence similarity with UPARSE (v10). Chimeras were detected and filtered with UCHIME. Taxonomy was assigned based on the UNITE (v8.2) database for fungi and SILVA (SSU138.1) for bacteria using uclust, with an 80% confidence threshold. The sequencing data are accessible in the NCBI SRA under BioProject PRJNA1301283.

Following the generation of the OTU abundance table, data normalization was performed to mitigate the influence of varying sequencing depths. Firstly, rarefaction was conducted by subsampling all samples to the minimum sequence depth to ensure fairness in subsequent alpha and beta diversity analyses. Furthermore, for downstream analyses that are sensitive to compositionality (such as LEfSe and PERMANOVA), the raw OTU table was transformed into relative abundance data.

### 2.5. Leaf Litter Extracellular Enzyme Stoichiometry (EES)

To assess microbial resource acquisition strategies in relation to carbon (C), nitrogen (N), and phosphorus (P), we applied a stoichiometric vector approach based on the activities of four key exoenzymes: β-glucosidase (*BG*) for C acquisition, N-acetyl-β-glucosaminidase (*NAG*) and leucine aminopeptidase (*LAP*) for N acquisition, and acid phosphatase (*AP*) for P acquisition [50,51]. Vector length, a dimensionless metric, reflects the degree of C limitation relative to N and P, with greater values indicating stronger C limitation. Vector angle (°) indicates whether microbial metabolism is more constrained by P (angle > 45°) or by N (angle < 45°) [52,53,54]. The two measures were calculated as follows:

$$\begin{array}{l} \mathrm{Vector}\;\mathrm{length}=\sqrt[{2}]{[\frac{Ln(BG)}{Ln(NAG+LAP)}{]}^{2}+[\frac{Ln(BG)}{Ln(AP)}{]}^{2}}\\ \mathrm{Vector}\;\mathrm{angle}=\mathrm{Degrees}\left\{ATAN2\left[\frac{Ln(BG)}{Ln(AP)}\right],\left[\frac{Ln(BG)}{Ln(NAG+LAP)}\right]\right\} \end{array}$$

In the expressions, ATAN2 represents the angle of the arc tangent from the origin to the point (*Ln* (*BG*)/*Ln* (*AP*), *Ln* (*BG*)/*Ln* (*NAG* + *LAP*)), and “Degrees” converts the result from radians to degrees.

### 2.6. Leaf Litter Organic Matter Quality

To evaluate the quality of leaf litter organic matter, Fourier-transform infrared (FTIR) spectroscopy was employed to quantify the relative degree of decomposition and recalcitrance using specific spectral band ratios [55]. Briefly, sample-KBr mixtures (1:100) were prepared and analyzed using a Fourier transform infrared spectrometer (FTIR, Shimadzu, Kyoto, Japan). Spectral acquisition was performed at 4 cm^−1^ resolution across 32 accumulative scans from 4000 to 400 cm^−1^, using pure KBr as background [56]. Two indices were derived from characteristic absorption bands associated with key molecular vibrations: polysaccharide C–O (oxygen-containing groups), aliphatic C–H, aromatic C=C, and aromatic C–H (carbon-rich groups). Index I (aromatic-to-aliphatic ratio) serves as an indicator of decomposition extent, with higher values reflecting advanced decomposition [52,55]. Index II, defined as the ratio of carbon-rich (aromatic and aliphatic) to polysaccharide-associated (oxygen-containing) bands, correlates positively with litter recalcitrance and reduced biological availability [52]. The formulas for both indices are given below:

$$\begin{array}{l} IndexI=\frac{RAISB_{aromatic\;C=C+aromatic\;C-H}}{RAISB_{aliphatic\;C-H}}\\ IndexII=\frac{RAISB_{aliphatic\;C-H+aromatic\;C=C+aromatic\;C-H}}{RAISB_{polysaccharide\;C-O}} \end{array}$$

In Index I, the numerator and denominator correspond to the relative areas of the infrared absorption bands (RAISBs) of aromatic and aliphatic C–H groups, respectively. For Index II, the numerator represents the combined RAISB of aromatic and aliphatic carbon groups, while the denominator refers to the RAISB of polysaccharide C–O bands.

### 2.7. Statistical Analysis

We employed one-way analysis of variance (ANOVA) to assess significant differences among treatments for mass loss, microbial respiration, enzyme activity, organic matter quality, enzyme vector angles, enzyme vector lengths, and microbial connectivity. When ANOVA assumptions (normality and homogeneity of variances) were satisfied, significant differences between individual treatments were further analyzed using Duncan’s multiple comparison test. In cases where assumptions were not fully met, appropriate data transformations (e.g., log, square-root) were applied prior to analysis. The Kruskal–Wallis test was used to evaluate differences in microbial alpha diversity and community assembly among treatments, with post hoc Dunn’s test identifying specific pairwise differences. A significance level of *p* < 0.05 was applied to all analyses, which were conducted in R version 4.3.3.

#### 2.7.1. Microbial Diversity

We calculated the alpha diversity indices (including Chao1, ACE, Shannon and Simpson) of bacteria and fungi using OTU richness data. Microbial beta diversity was assessed using Principal Coordinate Analysis (PCoA), based on standardized OTU data to calculate Bray–Curtis distances. To assess the differences in microbial community composition, we conducted a multivariate analysis of variance (PERMANOVA) based on Bray–Curtis distances using the cal_manova function. To ensure that the observed effects were not driven by differences in group dispersions rather than centroid locations, we also performed a test for homogeneity of multivariate dispersions (PERMDISP) prior to interpreting the PERMANOVA results. These analyses were conducted using the microeco package [57] in R software version 4.3.3.

#### 2.7.2. Microbial Community Assembly

To assess the mechanisms driving microbial community assembly during leaf litter decomposition, we applied the null-model framework established by Stegen et al. [35]. Based on both phylogenetic and abundance information, the beta Nearest Taxon Index (βNTI) and Raup–Crick index (RCbray) were computed using the microeco package in R. These metrics were used to evaluate the relative influence of deterministic versus stochastic processes governing community turnover.

The assembly processes were categorized according to thresholds derived from βNTI and RCbray values [35,58,59]. |βNTI| > 2 suggests a dominant role of deterministic selection, with βNTI < −2 indicating homogeneous selection and βNTI > +2 reflecting heterogeneous selection. When |βNTI| < 2 and |RCbray| > 0.95, dispersal processes prevail: RCbray < −0.95 corresponds to homogenizing dispersal, and RCbray > +0.95 implies dispersal limitation. Compositional changes were attributed to drift when both |βNTI| < 2 and |RCbray| < 0.95. Through inter-treatment comparisons of βNTI and RCbray, the contributions of selection, dispersal, and drift to community assembly were quantified.

#### 2.7.3. Co-Occurrence Network Analysis

To minimize spurious correlations, fungal and bacterial OTUs with relative abundances below 0.01% or occurring in fewer than 80% of samples were excluded from network construction, following established practices [60]. For each treatment, pairwise Spearman rank correlations were computed among all OTUs, with *p*-values adjusted using the false discovery rate (FDR) method. Only correlations with |ρ| ≥ 0.8 and FDR-adjusted *p* < 0.01 were considered biologically relevant and retained for subsequent analysis [60]. Undirected co-occurrence networks were constructed using the microeco package in R. The topological properties of each network were quantified, and networks were visualized in Gephi (version 0.10.1) using the Fruchterman–Reingold layout algorithm [61]. To identify key microbial taxa, within-module connectivity (Zi) and among-module connectivity (Pi) were computed with the igraph package. Based on established thresholds [62,63], nodes were categorized into four topological roles: peripheral (Zi < 2.5 and Pi < 0.62), connectors (Zi < 2.5 and Pi ≥ 0.62), module hubs (Zi ≥ 2.5 and Pi < 0.62), or network hubs (Zi ≥ 2.5 and Pi ≥ 0.62). Keystone taxa—defined as connectors, module hubs, and network hubs—were identified according to the criteria described in Guimerà and Amaral [61] and Tang et al. [63].

Network robustness was assessed using the robustness class within the meconetcomp package in R. The analysis incorporated multiple strategies for the removal of edges and nodes [64,65]. Edge removal strategies included: “edge_rand” (random removal) and “edge_strong” (removal in decreasing order of edge weight). Node removal strategies included: “node_rand” (random removal) and “node_degree_high” (removal in decreasing order of node degree). These strategies were evaluated against three robustness measures: “Eff” (network efficiency), “Eigen” (natural connectivity), and “Pcr” (the critical removal fraction required for network disintegration).

A detailed description of edge and node removal strategies and robustness measures can be seen in the study of Liu et al. [65].

The cohesion was implemented in cohesion class of the meconetcomp package in R for quantifying the connectivity of microbial communities. The detailed definition of cohesion was seen in the study of Herren and McMahon [66].

#### 2.7.4. LEfSe Analysis

To identify differentially abundant microbial taxa across treatment groups, we performed LEfSe (Linear discriminant analysis Effect Size) analysis following the approach described by Segata et al. [67]. The analysis was carried out using the microeco package in R.

The LEfSe algorithm first identifies features with significant abundance differences between groups through the Kruskal–Wallis rank-sum test (*p* < 0.01). Subsequently, pairwise comparisons are conducted using the Wilcoxon rank-sum test to ensure the consistency of differential abundance. Finally, linear discriminant analysis (LDA) is applied to estimate the effect size of each significantly enriched taxon, with an LDA score threshold of |LDA| > 3.0 applied to identify robust biomarkers [68,69].

## 3. Results

### 3.1. Mass Loss, Microbial Respiration and Organic Matter Quality

**Figure 2:**
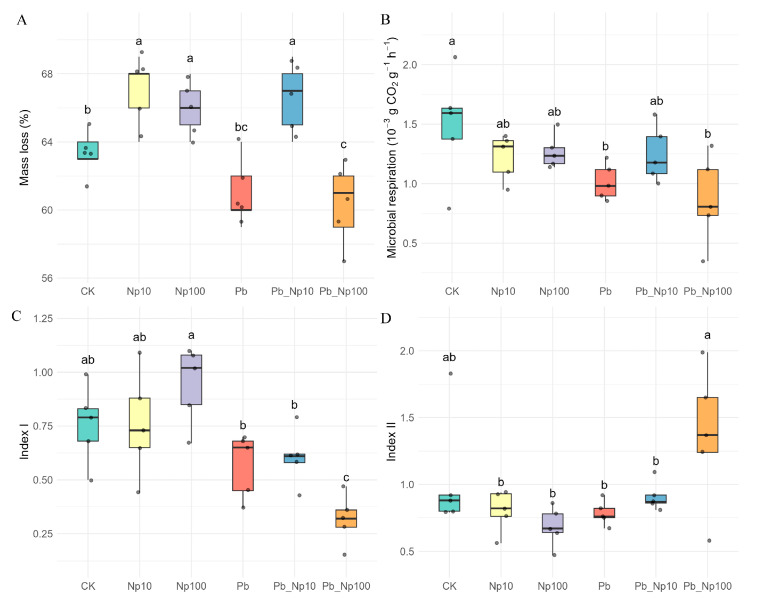
Differences in mass loss (**A**), microbial respiration (**B**), and organic matter quality (IndexI (**C**) and IndexII (**D**)) at the end of litter decomposition under different treatments. Different lowercase letters denote statistically significant differences (*p* < 0.05, Duncan’s test) among treatments.

After a 90-day litter decomposition experiment, lead addition alone suppressed decomposition relative to the control, although the difference was not statistically significant (mass loss: 61.0% for Pb vs. 63.2% for CK; Figure 2A). In contrast, nanoplastic (NP) addition alone significantly elevated mass loss (67.0%, *p* < 0.05). Under combined exposure, the Pb_NP10 treatment significantly increased mass loss (66.6%, *p* < 0.05), whereas the Pb_NP100 treatment significantly reduced it (60.4%, *p* < 0.05). For microbial respiration, Pb and NP individually, as well as their combined treatments, all exerted inhibitory effects (Figure 2B), with the lowest rate observed in Pb_NP100 (0.87 × 10^−3^ g CO_2_ g^−1^ h^−1^).

The response of Index I to Pb, NP, and their combined exposures mirrored the mass-loss pattern (Figure 2C): Pb alone decreased Index I, NP alone increased Index I, and the combined treatments led to a reduction in Index I. For Index II, values overall did not differ significantly from the control (Figure 2D); however, Index II in the Pb_NP100 treatment was significantly higher than under either Pb or NP alone.

### 3.2. Extracellular Enzyme Activity and Stoichiometry

As shown in Figure S1, enzyme activities related to litter decomposition displayed distinct treatment responses. Pb alone did not significantly influence exocellulase activity (Figure S1A), whereas sole NP addition significantly suppressed this enzyme (*p* < 0.05). Under combined exposure, only Pb_NP10 significantly elevated exocellulase activity (*p* < 0.05). For endocellulase (Figure S1B), both Pb and NP individually significantly increased activity, while combined exposure significantly enhanced activity solely in Pb_NP10. β-Glucosidase activity (Figure S1C) was significantly increased only by NP in the NP10 treatment, with no significant effects observed in the remaining treatments. Laccase and peroxidase (Figure S1D,E) responded similarly: both enzymes were stimulated by sole Pb or NP but inhibited under combined exposure. β-N-Acetylglucosaminidase activity (Figure S1F) was enhanced by Pb alone but suppressed by high-concentration NP (100 μg/L); combined exposure significantly increased activity exclusively in Pb_NP10. Leucine aminopeptidase activity (Figure S1G) was significantly elevated by all treatments, including sole Pb, sole NP, and combined exposures. Conversely, acid phosphatase activity (Figure S1H) was unaffected across all treatments.

**Figure 3:**
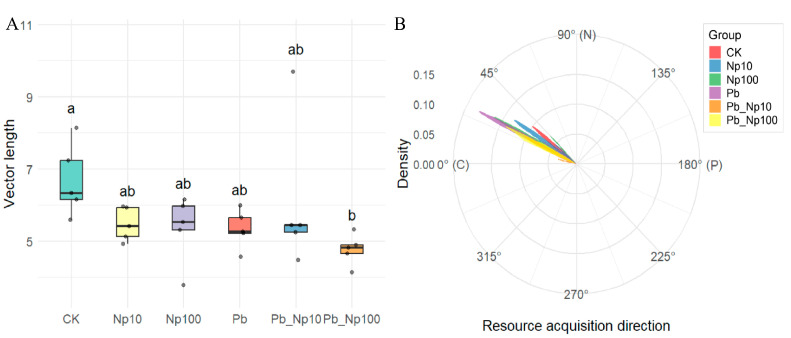
Differences in vector length (**A**) and vector angle (**B**) at the end of litter decomposition under different treatments. Different lowercase letters denote statistically significant differences (*p* < 0.05, Duncan’s test) among treatments.

Enzyme stoichiometry vectors during litter decomposition exhibited distinct characterization across treatments (Figure 3). Vector lengths were substantially greater than 1 under all treatments. Although Pb or NP addition alone reduced vector length values, these reductions were not statistically significant. In contrast, combined exposure (Pb_NP100) significantly decreased vector length (Figure 3A). Vector angle analysis revealed angles below 45° for all treatments, indicating that nitrogen limitation governed decomposition (Figure 3B). Moreover, Pb alone, NP alone, and their combined exposures all reduced vector angles, thereby intensifying nitrogen limitation.

### 3.3. Microbial Community Structure and Diversity

**Figure 4:**
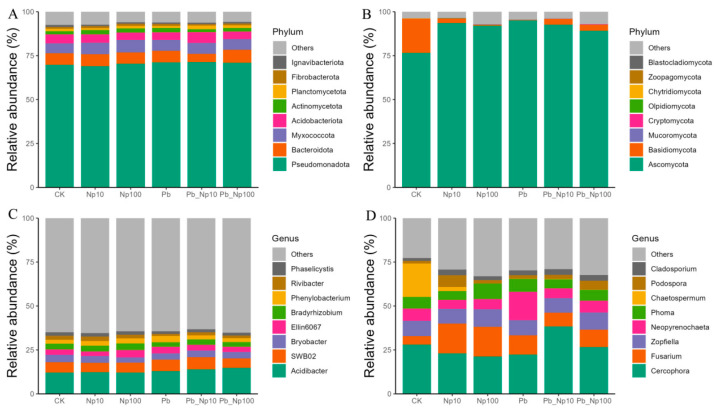
The relative abundances (%) of bacteria (**A**,**C**) and fungi (**B**,**D**) at the phylum and genus levels at the end of the litter decomposition under different treatments.

At the phylum level, *Pseudomonadota* overwhelmingly dominated the bacterial community across all treatments, and no significant differences were observed in relative abundance profiles among treatments (Figure 4A). Among fungi, *Ascomycota* was the dominant phylum. In the control (CK), *Basidiomycota* also constituted a substantial proportion (>20%); however, treatments with Pb, NP, or their combined exposures significantly reduced the relative abundance of *Basidiomycota* (Figure 4B). At the genus level, *Acidibacter* predominated in the bacterial community. Consistent with the phylum-level pattern, bacterial genus-level relative abundances showed minimal variation across treatments (Figure 4C). Conversely, the fungal community, primarily dominated by the genus *Cercophora* in all treatments, exhibited considerably greater variation among treatments. Specifically, all experimental treatments significantly increased the relative abundance of *Fusarium* while markedly decreasing that of *Chaetospermum* relative to the control (Figure 4D).

**Figure 5:**
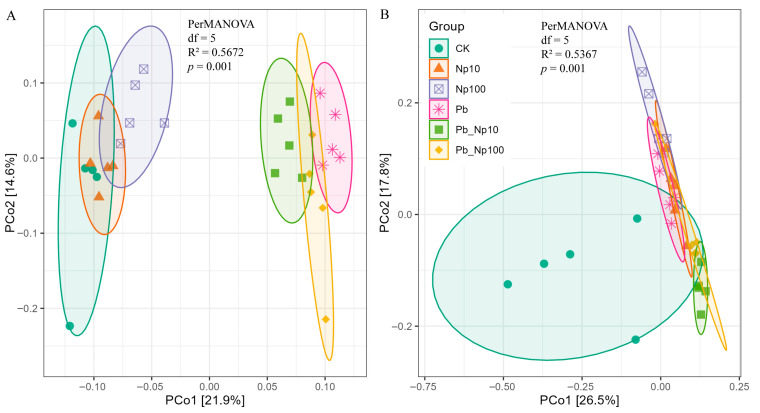
Principal coordinates analysis (PCoA) for bacterial (**A**) and fungal (**B**) communities at the OTU level based on the Bray–Curtis distance at the end of litter decomposition under different treatments.

PCoA revealed that both bacterial and fungal communities at the end of litter decomposition exhibited significant structural divergence among treatments (*p* < 0.01), indicating distinct compositional shifts in the decomposer community (Figure 5). For bacteria, Pb addition induced a clear separation in community structure (Figure 5A); for fungi, all treatments deviated significantly from the control (Figure 5B).

**Figure 6:**
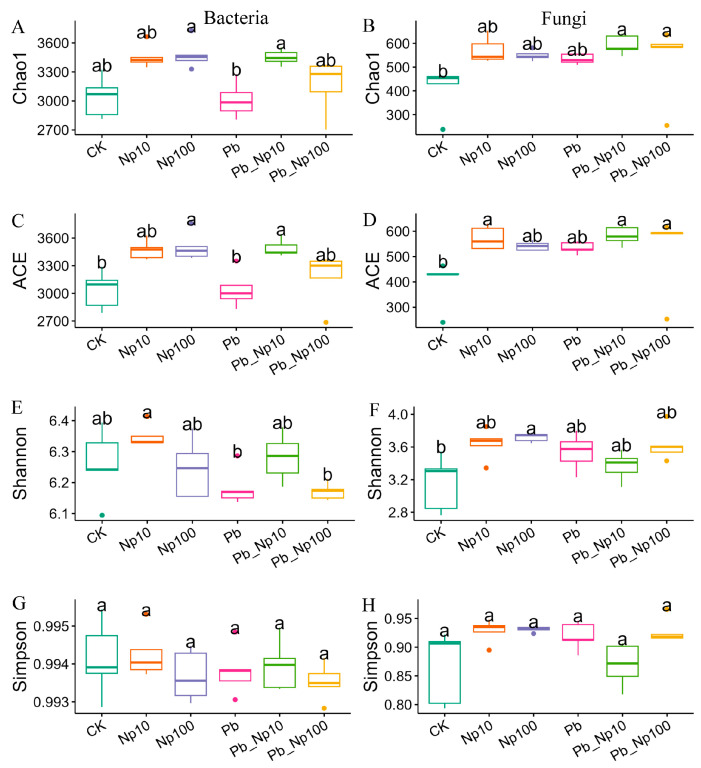
The alpha diversity (Chao1, ACE, Shannon and Simpson) of bacterial (**A**,**C**,**E**,**G**) and fungal (**B**,**D**,**F**,**H**) communities at the end of litter decomposition under different treatments. Different lowercase letters denote statistically significant differences (*p* < 0.05, Dunn’s test) among treatments.

As shown in Figure 6, alpha-diversity indices at the end of the decomposition revealed differences among treatments. The Chao1 index indicated elevated diversity in both bacteria and fungi across most treatments relative to the control, though these increases were statistically insignificant (Figure 6A,B). A largely consistent response was evident for the ACE index (Figure 6C,D). Regarding the Shannon index, bacterial communities exhibited no significant differences between treatments and the control in the vast majority of cases (Figure 6E). For fungi, most treatments displayed a trend toward higher diversity, yet this did not reach statistical significance (Figure 6F). Neither bacterial nor fungal communities showed significant differences in the Simpson index across any treatments (Figure 6G,H).

**Figure 7:**
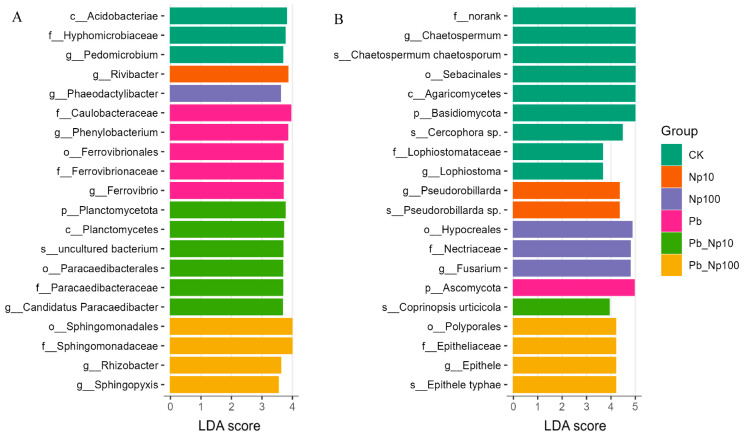
LEfSe analysis of bacterial (**A**) and fungal (**B**) communities at the end of litter decomposition under different treatments.

Combined with the ANCOM-BC model, the LEfSe analysis of microbial communities (Figure 7) identified 20 bacterial clades and 20 fungal clades that showed statistically significant differences among treatments (with an LDA score threshold > 3.0). Each treatment harbored distinct clades at peak abundance, demonstrating that Pb, nanoplastic (NP), and their co-exposure drove taxonomic differentiation across hierarchical levels. For bacteria, *Acidobacteriae* (class), *Hyphomicrobiaceae* (family), and *Pedomicrobium* (genus) dominated in controls; *Phaeodactylibacter* (genus) peaked under NP100; *Caulobacteraceae* (family), *Phenylobacterium* (genus), *Ferrovibrionales* (order), *Ferrovibrionaceae* (family), and *Ferrovibrio* (genus) reached maximal abundance in Pb treatments; whereas *Sphingomonadales* (order), *Rhizobacter* (genus), and *Sphingopyxis* (genus) were enriched under Pb_NP100 co-exposure. For fungi, *Hypocreales* (order), *Nectriaceae* (family), and *Fusarium* (genus) prevailed in NP100; *Ascomycota* (phylum) dominated Pb treatments; *Polyporales* (order), *Epitheliaceae* (family), *Epithele* (genus), and *Epithele typhae* (species) exhibited the highest abundance under Pb_NP100 co-exposure.

### 3.4. Co-Occurrence Network Analysis

**Figure 8:**
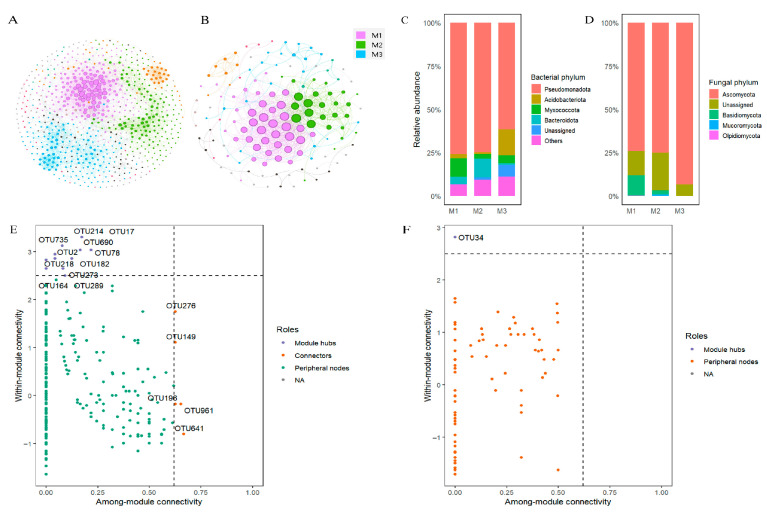
Microbial co-occurrence networks ((**A**) for bacteria and (**B**) for fungi), the relative abundance of modules at phylum level ((**C**) for bacteria and (**D**) for fungi) and keystone taxa identified through network analysis ((**E**) for bacteria and (**F**) for fungi) at the end of litter decomposition.

Co-occurrence network analysis revealed substantially higher complexity in bacterial networks than in fungal networks across all samples (Figure 8A,B). This differential complexity was further reflected in the composition of the top three network modules, where bacterial phyla markedly outnumbered fungal taxa within each module. In bacterial networks, *Pseudomonadota* overwhelmingly dominated all three modules, with *Myxococcota*, *Bacteroidota*, and *Acidobacteriota* emerging as secondary dominant phyla in Modules M1, M2, and M3, respectively (Figure 8C). Fungal networks exhibited absolute dominance of *Ascomycota* across all modules, with *Basidiomycota* additionally representing a substantial component in Module M1 (Figure 8D). Network topology analysis identified 16 bacterial keystone taxa versus only one fungal keystone taxon (Figure 8E,F; Table S1), collectively demonstrating more complex network characteristics in bacterial communities relative to fungal communities. The number of keystone taxa did not differ significantly across treatments (Table S1), indicating that Pb, NP, and their co-exposures exerted limited impact on core structural hubs within the networks.

**Figure 9:**
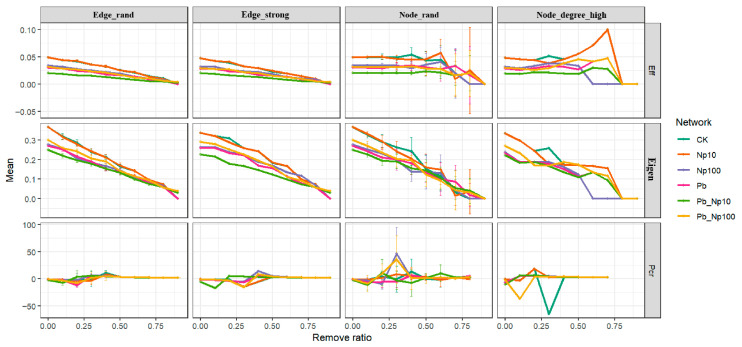
Four types of edge and node removal strategies (edge_rand, edge_strong, node_rand and node_degree_high) and three types of measure methods (Eff, Eigen and Pcr) for robustness analysis of bacterial network under different treatments.

Robustness analysis of bacterial co-occurrence networks (Figure 9) revealed that among the three assessment metrics, efficiency (Eff) and eigenvalue (Eigen) most distinctly captured treatment-induced patterns. All treatments—Pb, NP, and their co-exposure—significantly reduced bacterial network robustness, with the greatest decline observed under Pb_NP10 co-exposure. For fungal networks (Figure S2), the NP treatment was excluded from robustness assessment because it failed to meet network correlation thresholds; subsequent analysis showed that Pb and NP co-exposure significantly decreased fungal network robustness.

**Figure 10:**
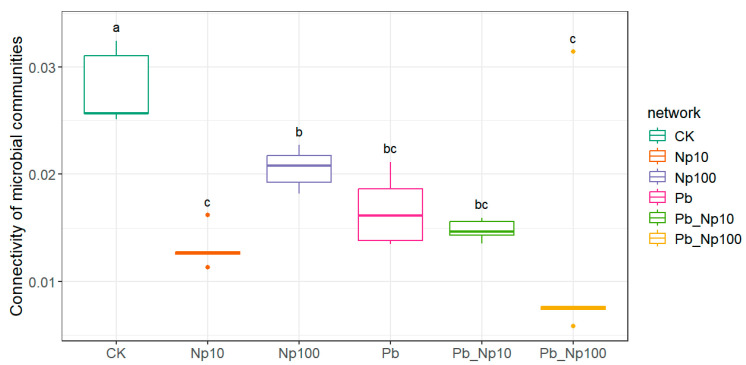
The connectivity of bacterial communities at the end of litter decomposition under different treatments. Different lowercase letters denote statistically significant differences (*p* < 0.05, Duncan’s test) among treatments.

As shown in Figure 10, both Pb, NP, and their co-exposure significantly reduced bacterial community connectivity, with the strongest effect under Pb_NP100 treatment. For fungal communities (Figure S3), connectivity assessment excluded the NP treatment because it failed to meet network correlation thresholds; subsequent analysis revealed no significant connectivity differences among the remaining treatments.

### 3.5. Community Assembly

**Figure 11:**
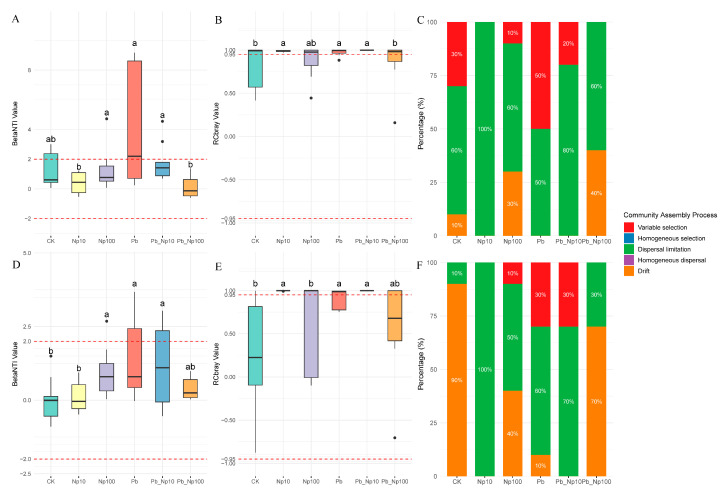
Microbial community assembly for bacteria (**A**–**C**) and fungi (**D**–**F**) at the end of litter decomposition under different treatments. Different lowercase letters indicate significant differences among different treatments (*p* < 0.05, Dunn’s test).

For both bacterial and fungal communities, the absolute values of nearly all βNTI metrics were below 2, indicating that stochastic processes predominantly governed community assembly (Figure 11A,D). RCbray values consistently exceeded −0.95, demonstrating the absence of homogeneous dispersal while highlighting the dominance of dispersal limitation and drift in stochastic assembly (Figure 11B,E). In bacterial communities, Pb addition alone enhanced the contribution of deterministic processes (variable selection), whereas NP alone increased stochastic processes; notably, combined exposures—particularly Pb_NP100—substantially amplified stochastic dominance. Conversely, in fungal communities, NP100, Pb, and Pb_NP10 treatments all elevated the proportion of deterministic processes.

## 4. Discussion

### 4.1. Litter Decomposition

Pb alone suppressed litter decomposition, as shown by reduced microbial respiration despite an insignificant decrease in mass loss, a pattern consistent with established findings [70,71]. In contrast, nanoplastic alone exerted concentration-dependent effects: NP10 (10 μg/L) markedly accelerated decomposition, while NP100 (100 μg/L) slightly inhibited it—aligning with the “biphasic effect” of NPs on litter decomposition reported in previous studies [72]. This facilitation at low NP concentrations, which contradicts some earlier reports [19,20,71] but echoes observations by Wang et al. [18], is tightly linked to enhanced cellulase activity: our data (Figure S1) show significantly elevated endocellulase and β-glucosidase activities in the NP10 group, likely driven by NP-mediated microbial aggregation. Tu et al. [73] proposed that such facilitation arises from altered microbial interactions, and we further clarify that low-concentration polystyrene NPs may adsorb extracellular polymers (EPS) secreted by cellulose-degrading taxa (e.g., Acidibacter, the dominant bacterial genus in our study; Figure 4C), promoting their colonization and synergistic cellulolytic activity [73]. Intriguingly, low-concentration NP–Pb co-exposure (Pb_NP10) also enhanced decomposition, whereas high-concentration co-exposure (Pb_NP100) suppressed it—illustrating a non-monotonic response that departs from linear concentration–effect models [38] yet supports the concentration-dependent regulation principle advanced by Seena et al. [72]. The inhibitory effect of high-concentration NPs in combination with Pb is associated with suppressed ligninolytic enzyme activity: laccase and peroxidase activities were significantly reduced in Pb_NP100 groups (Figure S1), potentially due to physical obstruction of litter surfaces by aggregated NPs or internalization into microbial cells, which disrupts the synthesis of lignin-degrading enzymes [19,20,74]. This biphasic pattern is not unique to our study: Seena et al. [72] also found that 10 μg/L PS-NPs promoted litter decomposition while 100 μg/L inhibited it, and the consistency likely stems from the high cellulose content of *M. floridulus* litter—rendering decomposition dynamics more sensitive to cellulase activity changes induced by low-concentration NPs, while high-concentration NPs target ligninolytic processes that become rate-limiting as decomposition proceeds. The parallel responses of mass loss and Index I (aromatic-to-aliphatic ratio) across treatments further indicate dominance of labile organic-matter (primarily cellulose) degradation, consistent with the substrate characteristics of *M. floridulus*. This mechanism is corroborated by concordant patterns in endocellulase, β-glucosidase, and ligninolytic enzyme activities, confirming their substantial contributions to decomposition dynamics.

Enzyme-stoichiometry-based resource-limitation analysis revealed that Pb–NP co-exposure reduced carbon limitation during decomposition. This reduction was likely due to inhibited microbial activity from co-exposure. As a result, abundant labile organic matter was preserved. The observed suppression of both microbial respiration and decomposition rates supports this hypothesis. Simultaneously, enzymatic metrics confirmed persistent nitrogen limitation throughout the process, with Pb–NP co-exposure intensifying this constraint: vector angles were consistently <45° across all treatments, and values further decreased with increasing pollution stress (Figure 3B), indicating a progressive shift toward stronger nitrogen limitation. This pattern aligns with the theory of enzyme stoichiometry proposed by Sinsabaugh et al. [50], which posits that a reduction in vector angle reflects microbial adaptive strategies to alleviate nitrogen scarcity—specifically, microbes downregulate carbon metabolic investment to allocate more resources to nitrogen acquisition. Our enzyme activity data further corroborate this mechanism: β-N-acetylglucosaminidase (NAG, a key nitrogen-mineralizing enzyme) activity was significantly reduced in the Pb_NP100 group compared to other treatments (Figure S1F), directly evidencing impaired nitrogen mineralization under high-concentration co-exposure. We propose a synergistic effect between Pb and NPs. Pb^2+^ likely inhibits catalytic activity by binding to the active sites of nitrogen-mineralizing enzymes [16]. Meanwhile, NPs reduce nitrogen source uptake by blocking microbial cell membrane channels [33]. This dual inhibition suppresses the conversion of organic nitrogen to bioavailable NH_4_^+^, intensifying inter-microbial competition for limited nitrogen resources. The attribution of heightened nitrogen limitation to NP internalization disrupting electron-transport systems [75] is thus complemented by these more specific mechanistic insights into enzyme inhibition and nutrient uptake obstruction.

### 4.2. Microbial Community Structure and Diversity

At the phylum level, bacterial community composition remained stable across all treatments. Pseudomonadota maintained a relative abundance over 60% in all groups (Figure 4A). This stability underscores deep functional redundancy within dominant lineages. This aligns with the “redundancy threshold effect” theory [76]. The pollutant concentrations did not exceed the threshold required to collapse core community functions. Specifically, our LEfSe analysis (Figure 7A) identifies taxon-specific replacement dynamics: Pb treatments enriched *Ferrovibrio* (a metal-tolerant iron-oxidizing genus), while Pb_NP100 treatments favored *Sphingopyxis* (a nanoplastics-resistant taxon), and both replaced sensitive cellulose-degrading taxa such as *Hyphomicrobium* (enriched in controls) [27]. These tolerant genera likely sustain cellulolytic function by upregulating metal transporter genes [27] and secreting detoxifying extracellular polymers, ensuring cellulose depolymerization remains unimpaired despite taxonomic turnover. This buffering capacity is further evidenced by the persistence of 16 bacterial keystone taxa in co-occurrence networks (Figure 8E), which anchor core ecological services. Fungi, by contrast, displayed pronounced vulnerability, rooted in a clear “taxon-function” disconnect: *Basidiomycota*, which accounted for >20% of the fungal community in controls (Figure 4B), declined sharply under all pollutant regimes and was undetectable as an LEfSe biomarker in treated groups (Figure 7B). As the primary lignin-degrading phylum, its loss directly explains the suppression of ligninolytic enzymes (laccase and peroxidase; Figure S1D,E) observed in polluted treatments. Compounding this, the fungal community was dominated by a single keystone taxon (*Fusarium* sp., *Ascomycota*; Figure 8F), which possesses limited cellulolytic ability but lacks lignin-degrading capacity [19]—resulting in functional monotony and heightened vulnerability. Ecologically, this fragility stems from fungi’s smaller effective population size (typically 10–100-fold lower than bacteria) [35], making sensitive taxa like *Basidiomycota* more prone to extinction via ecological drift under pollutant stress.

Despite phylum-level inertia, OTU-based beta-diversity revealed significant compositional divergence for both kingdoms (Figure 5), indicating that pollutants suppress specialized, low-abundance taxa or guilds rather than entire phyla. Zhang et al. [77] observed a marked decline in nitrogen-cycling genera such as *Nitrospira* and *Terrimonas* under NP exposure. In our study, pollutant addition intensified nitrogen limitation, corroborating the suppression of nitrogen-cycling taxa. This pattern of taxonomic persistence coupled with functional turnover aligns with microbial successional hysteresis [78], wherein rapid strain-level adaptations shield higher-level taxonomic structure while simultaneously re-engineering ecosystem function. Furthermore, it mirrors observations from polluted systems, where pollutant-tolerant bacterial genera (e.g., *Rivibacter*, *Phaeodactylibacter*, *Phenylobacterium*, *Ferrovibrio*, *Candidatus Paracaedibacter*, *Rhizobacter*, *Sphingopyxis*) and fungi (e.g., *Pseudorobillarda*, *Fusarium*, *Epithele*) become dominant without altering phylum-level dominance [27].

The frequent, albeit statistically non-significant, increases in microbial diversity observed under NP and NP–Pb co-exposure further complicate the narrative, diverging from reports of diversity suppression [41,79]. We interpret these transient gains as hormetic responses rooted in microhabitat and metabolic effects. Specifically, low-concentration NP (10 μg/L) likely adsorbs labile organic carbon, trace metals, or extracellular polymers from the environment to form micro-niches [80], which stimulate the proliferation of r-strategists such as *Sphingomonas*—this offsets the loss of sensitive taxa, thereby maintaining alpha diversity. This phenomenon of low contaminant doses driving such stimulatory effects is well documented in previous studies [80,81]. At higher NP concentrations, while more taxa are inhibited, the enrichment of pollution-tolerant genera (e.g., *Fusarium*; Figure 4D) prevents a sharp decline in diversity. Importantly, this hormetic pattern reflects enhanced metabolic diversity via the mild activation of microbial stress responses [81], rather than a true increase in species richness. However, such effects typically wane as toxicity accumulates over longer incubations or at higher concentrations, ultimately leading to diversity decline.

### 4.3. Microbial Co-Occurrence Network and Community Assembly

In bacterial networks, *Pseudomonadota* emerged as the absolutely dominant phylum across all treatments and overwhelmingly dominated the top three functional modules, underscoring its pivotal role as a key functional group. Bacterial networks exhibited significantly greater complexity than fungal networks: the top three modules contained markedly higher phylum diversity, and 16 keystone taxa (nearly half belonging to *Pseudomonadota*; Figure 8E) were identified—far more than in fungal networks—indicating stronger interspecies associations. Crucially, these keystone taxa maintained stable identity and abundance across treatments, and bacterial network connectivity, though slightly reduced in the Pb_NP100 group, remained above 0.015 (Figure 10)—a threshold that preserves network resilience. High connectivity facilitates frequent metabolite exchange (e.g., cross-feeding) among microbes, and even if some taxa are inhibited, keystone taxa can sustain network structure via “redundant connections” [82]. The stability of these keystone taxa likely stems from their possession of multi-drug resistance genes and broad metabolic capacities, enabling them to persist under pollutant stress. This robustness mitigates cascading extinctions of “critical nodes” and ensures efficient material and energy flows [82], while consistent keystone taxa imply that core functions—cellulose hydrolysis, nitrification, and denitrification—were continually driven by resilient bacterial strains capable of compensating for sensitive taxa [76,83]. These results confirm that bacterial communities retained structural stability and functional redundancy, with no collapse-like disruptions observed under any treatment.

By contrast, fungal networks displayed markedly lower complexity: fewer phyla were represented in the top three modules, only one OTU was identified as a keystone taxon across all treatments (Figure 8F), and high modularity was observed, indicating strong functional differentiation but weak inter-module cooperation. As Liu et al. [65] noted, high modularity reduces network resilience, as the loss or inhibition of a single keystone taxon can trigger cascading disruptions. Indeed, in some pollutant treatments, stability and connectivity models could not be constructed owing to correlations below the required threshold, and stability analyses revealed collapse-like declines under certain regimes. Collectively, these findings demonstrate that bacteria—not fungi—are the primary functional decomposers of litter in this riverine ecosystem, aligning with observations in many aquatic systems [84,85,86].

Assembly processes of both fungal and bacterial communities involved in litter decomposition were almost entirely governed by stochasticity, with βNTI absolute values mostly <2 (Figure 11A,D)—a pattern driven by the laboratory microcosm setting. The homogeneous environmental conditions (constant temperature, light, and pollutant dosing) weakened deterministic environmental filtering, while the 125 rpm shaking regime simulated gentle flow to promote microbial dispersal—reducing dispersal limitation and elevating ecological drift as the dominant stochastic process [35]. Although Pb exposure slightly increased the deterministic component (evidenced by marginally higher βNTI values in Pb-only groups), functional redundancy within *Pseudomonadota* (e.g., tolerant taxa replacing sensitive ones without disrupting core functions) weakened the impact of this shift, ensuring stochasticity remained dominant (>50% contribution). This aligns with the prevalence of stochasticity in subtropical riverine systems, where frequent hydrological disturbances drive “ecological drift + dispersal limitation” [87,88,89], but our controlled conditions amplified drift by minimizing dispersal barriers. Despite these assembly differences between kingdoms, ecosystem functioning remained unimpaired: highly redundant bacterial (e.g., *Pseudomonadota*) and fungal (e.g., *Ascomycota*) taxa rapidly compensated for sensitive members, sustaining carbon and nitrogen transformation efficiency. A vector angle below 45° directly indicates the absence of phosphorus limitation. While subtropical ecosystems are generally P-limited, soluble orthophosphate inputs from domestic wastewater and agricultural runoff (detergents, livestock manure) in Jishou City likely alleviated this constraint, as microbes require only minimal external phosphorus to overcome P limitation.

## 5. Conclusions

This study demonstrates that Pb and NP contamination differentially influence litter decomposition and microbial community dynamics in subtropical riverine ecosystems. While Pb alone inhibited microbial respiration and slowed decomposition, NP exhibited concentration-dependent effects—accelerating decomposition at low concentrations (likely through modified microbial interactions) but suppressing it at high levels. The non-monotonic response under Pb-NP co-exposure highlights the complexity of pollutant interactions, where synergistic or antagonistic effects depend on contaminant dosage.

Microbial community analysis revealed that bacterial assemblages, dominated by functionally redundant taxa like *Pseudomonadota*, maintained structural and network stability despite pollutant stress. Their high connectivity, keystone taxon persistence, and physiological resilience ensured continued organic matter processing (e.g., cellulose hydrolysis) under Pb, NP, or combined exposure. In contrast, fungal communities displayed pronounced vulnerability, with reduced diversity, collapsed network stability, and near-elimination of key decomposers like *Basidiomycota*—confirming bacteria as the primary drivers of litter decomposition in this system.

Stochastic processes overwhelmingly governed microbial assembly, with pollutants only marginally enhancing deterministic selection. This stochastic dominance, coupled with high functional redundancy, buffered ecosystem functions against contamination-induced disturbances. Notably, enzymatic and stoichiometric analyses revealed that Pb-NP co-exposure alleviated carbon limitation (via reduced microbial activity) but exacerbated nitrogen limitation, likely due to disrupted nitrogen mineralization pathways.

Our findings underscore the resilience of bacterial-mediated decomposition in contaminated aquatic ecosystems, though long-term pollutant exposure may erode this stability if fungal functional collapse persists. Future studies should investigate legacy effects of sequential or chronic contamination on microbial succession and nutrient cycling. Additionally, the hormetic stimulation of microbial diversity under low NP doses warrants further exploration, as transient diversity increases may mask accumulating toxicity risks.

## Data Availability

The raw sequencing reads generated in this study have been deposited in the NCBI Sequence Read Archive (SRA) under the BioProject accession number PRJNA1301283.

## Supplementary Materials

The following supporting information can be downloaded at https://www.mdpi.com/article/10.3390/microorganisms13092172/s1. Figure S1: Changes in the activity of single extracellular enzymes at the end of litter decomposition under different treatments; Figure S2: Four types of edge and node removal strategies (edge_rand, edge_strong, node_rand and node_degree_high) and three types of measure methods (Eff, Eigen and Pcr) for robustness analysis of fungal network; Figure S3: The connectivity of fungal communities at the end of litter decomposition under different treatments. c_pos denotes the connectivity of microbial communities. Same lowercase letters denote statistically no-significant differences (*p* > 0.05, Duncan’s test) among treatments; Table S1: Keystone taxa identified from co-occurrence network under different treatments.
