# Supplementary material for: Impacts of Lead and Nanoplastic Co-Exposure on Decomposition, Microbial Diversity, and Community Assembly Mechanisms in Karst Riverine Miscanthus Litter
Source: Microorganisms. 2025 Sep 17;13(9):2172. doi: 10.3390/microorganisms13092172 (PMC12472299; doi:10.3390/microorganisms13092172)
Supplement: Supplementary file 1 [file microorganisms-13-02172-s001.zip › microorganisms-3842970-supplementary.pdf]

Supplementary Materials:

Impacts of Lead and Nanoplastic Co-Exposure on Decomposition, Microbial Diversity, and Community Assembly Mechanisms in Karst Riverine *Miscanthus* Litter

Peijian Chen [1,2], Tianjiao Mei [1,2], Xingbing He [1,2,*], Yonghui Lin [1,2,*], Zaihua He [1,2] and Xiangshi Kong [3]

1 College of Biology and Environmental Sciences, Jishou University, Jishou 416000, China

2 Hunan Provincial key Laboratory of Ecological Conservation and Sustainable Utilization of Wulingshan Resources, Jishou University, Jishou 416000, China

3 College of Tourism and Management Engineering, Jishou University, Zhangjiajie 427000, China

* Correspondence: hexb@jsu.edu.cn or hexb2004@163.com (X.H.); linyonghui@jsu.edu.cn (Y.L.)

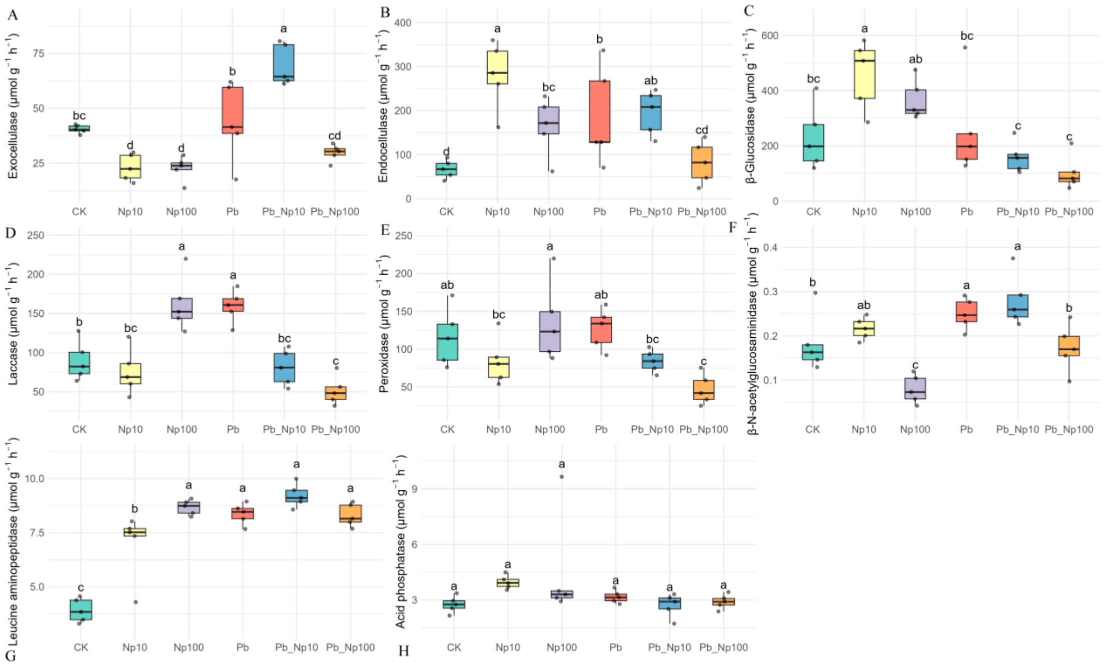

Figure S1. Changes in the activity of single extracellular enzymes at the end of litter decomposition under different treatments.

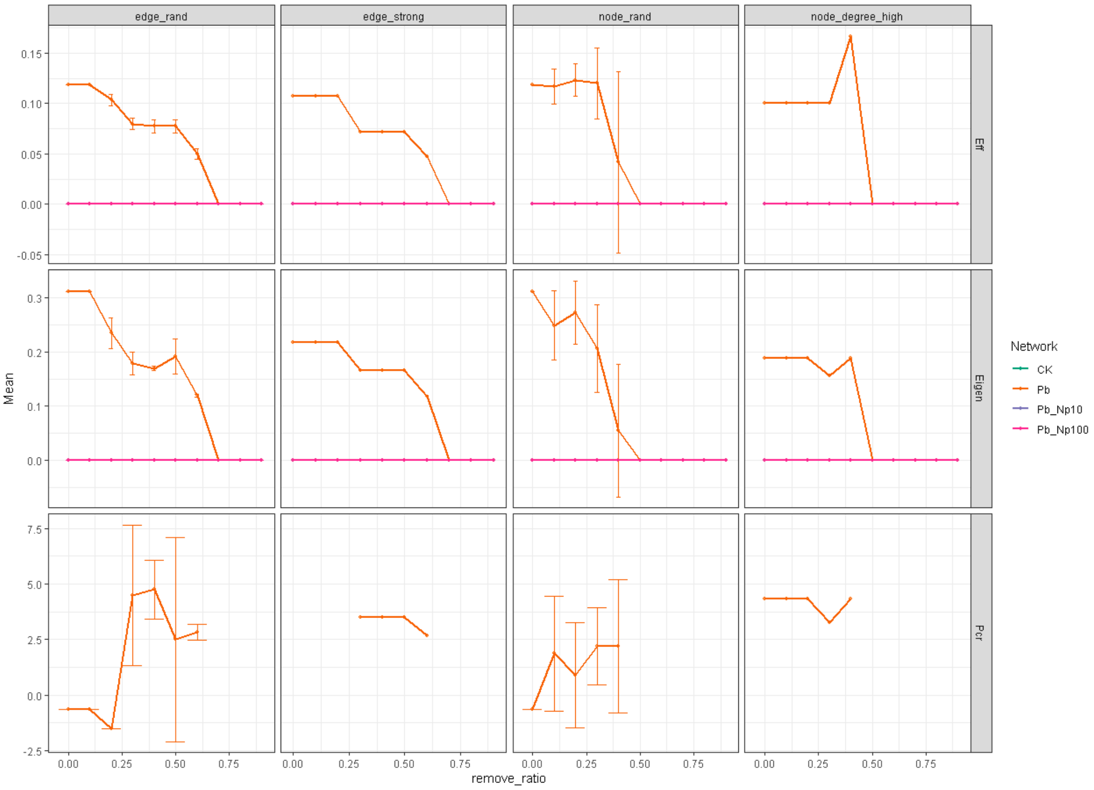

Figure S2. Four types of edge and node removal strategies (edge_rand, edge_strong, node_rand and node_degree_high) and three types of measure methods (Eff, Eigen and Pcr) for robustness analysis of fungal network.

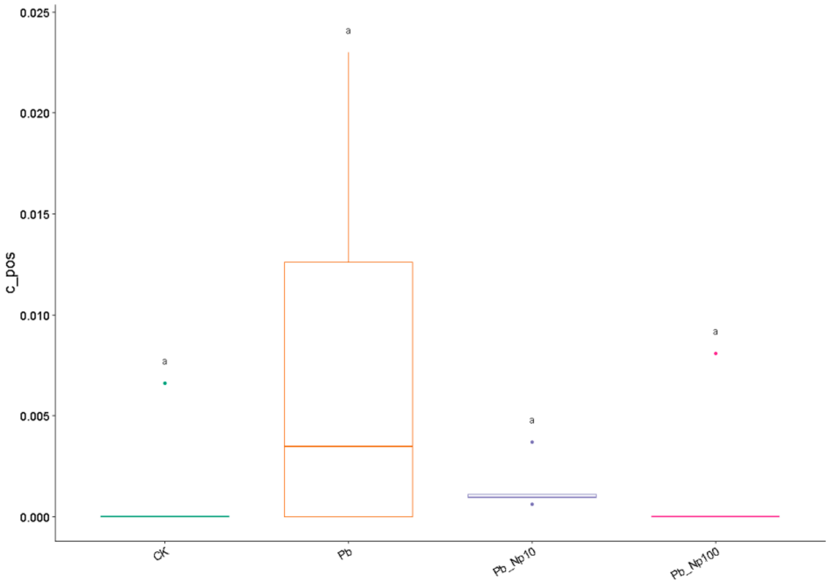

Figure S3. The connectivity of fungal communities at the end of litter decomposition under different treatments. c_pos denotes the connectivity of microbial communities. Same lowercase letters denote statistically no-significant differences ($p > 0.05$, Duncan's test) among treatments.

Table S1 Keystone taxa identified from co-occurrence network under different treatments

Keystone Taxa	Species	Genus	Phylum	CK	Np10	Np100	Pb	Pb_Np10	Pb_Np100
b_OTU17	unidentified	Pedomicrobium	Pseudomonadota	+	+	+	+	+	+
b_OTU78	unidentified	Caenimonas	Pseudomonadota	+	+	+	+	+	+
b_OTU690	unidentified	unidentified	Verrucomicrobiota	+	+	+	+	+	+
b_OTU182	unidentified	unidentified	unidentified	+	+	+	+	+	+
b_OTU273	unidentified	Hassallia	Bacteroidota	-	+	+	+	+	-
b_OTU289	unidentified	Phaselicystis	Myxococcota	+	+	+	+	+	+
b_OTU214	unidentified	Sphingobacterium	Bacteroidota	+	+	+	+	+	+
b_OTU735	unidentified	Candidatus Rhabdochlamydia	Chlamydiota	+	+	+	+	+	+
b_OTU2	unidentified	Acidibacter	Pseudomonadota	+	+	+	+	+	+
b_OTU218	unidentified	unidentified	Myxococcota	+	+	+	+	+	+
b_OTU164	unidentified	Terrimonas	Bacteroidota	+	+	+	+	+	+
b_OTU276	unidentified	unidentified	Pseudomonadota	+	+	+	+	+	+
b_OTU149	unidentified	Rhodoplanes	Pseudomonadota	+	+	+	+	+	+
b_OTU196	unidentified	Candidatus Jidaibacter	Pseudomonadota	+	+	+	+	+	+
b_OTU961	unidentified	Wenzhouxiangella	Pseudomonadota	+	+	+	+	+	+
b_OTU641	unidentified	Bryobacter	Acidobacteriota	+	+	+	+	+	+
F_OTU34	Fusarium sp.	Fusarium	Ascomycota	+	+	+	+	+	+
